# Serological Cross-Reactivity among *Orientia tsutsugamushi* Serotypes but Not with *Rickettsia japonica* in Japan

**DOI:** 10.3390/tropicalmed3030074

**Published:** 2018-07-05

**Authors:** Eiichiro Sando, Koya Ariyoshi, Hiromi Fujita

**Affiliations:** 1 Department of General Internal Medicine, Kameda Medical Center, Chiba 296-8602, Japan; 2 Department of Clinical Tropical Medicine, Nagasaki University Graduate School of Biomedical Sciences, Nagasaki 852-8523, Japan; kari@nagasaki-u.ac.jp; 3 Department of Clinical Medicine, Institute of Tropical Medicine (NEKKEN), Nagasaki University, Nagasaki 852-8521, Japan; 4 Mahara Institute of Medical Acarology, Tokushima 779-1510, Japan; fujitah7knu@y8.dion.ne.jp

**Keywords:** *Orientia tsutsugamushi*, *Rickettsia japonica*, scrub typhus, Japanese spotted fever, tsutsugamushi disease

## Abstract

The rickettsial diseases Japanese spotted fever (JSF) and scrub typhus (ST) are caused by *Rickettsia japonica* and *Orientia tsutsugamushi*, respectively. The diseases share clinical symptoms, such as fever, rash, and eschar. However, there are no systematical investigations of the serological cross-reactivity between *R. japonica* and *O. tsutsugamushi*. Also, the serological cross-reactivity among *O. tsutsugamushi* serotypes is still unclear. We analyzed 1406 cases tested by indirect immunoperoxidase assay using seven rickettsial antigens—one *R. japonica* and six *O. tsutsugamushi* serotypes—between 2003 and 2016 at two reference centers in Japan. Of these, 167 JSF and 190 ST cases were serologically diagnosed. None of the ST cases had a significant increase in IgM titers against *R. japonica*. Six JSF cases showed IgG titers of ≥40 against *O. tsutsugamushi*, but no IgG titer showed a significant elevation in the convalescent phase sample. We observed a substantial degree of cross-reactivity between *O. tsutsugamushi* serotypes. Cross-reactivity was significant among Karp, Hirano/Kuroki, and Kato types and between Gilliam and Irie/Kawasaki types in IgM, while the Shimokoshi type was less cross-reactive than the others. In conclusion, there is no serological cross-reaction between *R. japonica* and *O. tsutsugamushi*. The cross-reactivity among *O.* *tsutsugamushi* varies depending on serotypes.

## 1. Introduction

Rickettsiae, obligate intracellular bacteria grouped in the family Rickettsiaceae, have two genera—*Rickettsia* and *Orientia*. In Japan, both *Rickettsia japonica* and *Orientia tsutsugamushi* are endemic, causing Japanese spotted fever (JSF) and scrub typhus (ST, or tsutsugamushi disease), respectively. The number of ST cases, according to the national surveillance data, has remained steady, with an average annual number of cases of 419 between 2007 and 2016, whereas that of JSF cases has increased from 98 in 2007 to 276 in 2016 [1]. Both pathogens belong to the same family and cause clinically similar diseases, such as fever without localizing signs, rash, and eschar [1], which can mislead clinicians into believing that these organisms may have cross-reactivity when a diagnosis of dual infection is made on the basis of serological tests. A lack of serological cross-reaction with *O. tsutsugamushi* has been demonstrated in only a limited number of reports of JSF [2,3], and no study has systematically investigated the serological cross-reactivity between *R. japonica* and *O. tsutsugamushi* with a large sample size. Also, cross-reactivity among multiple serotypes of *O. tsutsugamushi* have not been systematically evaluated yet.

## 2. Materials and Methods

We retrospectively analyzed the serology results of referral cases to two reference centers for rickettsial diseases in Japan between 1 January 2003, and 31 December 2016, at Ohara Research Laboratory, Ohara General Hospital in Fukushima and Mahara Institute of Medical Acarology in Tokushima. We enrolled all cases which were tested by indirect immunoperoxidase assay (IP), using seven rickettsial antigens—the Aoki strain of *R. japonica* and six *O. tsutsugamushi* strains (namely, Kato, Karp, Gilliam, Irie, Hirano, and Shimokoshi). The Kato, Karp, and Gilliam strains are classic standard serotypes. The Irie, Hirano, and Shimokoshi strains are newly added standard serotypes of Irie/Kawasaki, Hirano/Kuroki, and Shimokoshi, respectively. The rickettsial strains were cultured in L929 cells and used as antigens. The antigen was suspended in 0.01 M phosphate-buffered saline (PBS), pH 7.2, containing 0.3% bovine serum albumin fraction V. The antigen, diluted to 100–200 rickettsial particles per field of a microscope (×40), was spotted on a slide, air-dried for 30 min, and then fixed in acetone for 10 min at room temperature. After drying, the slide-antigen was used immediately, otherwise stored at −20 °C. Patient sera were diluted two-fold from 1:40 to 1:40960 with PBS, and 0.01 mL of each dilution was applied to the spot of the antigen on the slide, incubated for 30 min at 36 °C in a humidified chamber, and washed twice for 5 min in PBS. Then, 0.01 mL of 1:100-diluted antihuman IgG or IgM rabbit serum (γ-chain specific or μ-chain specific, DAKO Corp., Glostrup, Denmark) was added to each spot, followed by an incubation for 30 min at 36 °C in the chamber and washed as described above. The slide was finally incubated for 5 min at room temperature in a freshly prepared enzyme substrate solution composed of 1 volume of 80% ethanol containing 0.2% 4-Cl-1-naphtol, 4 volumes of PBS, and 0.01 volume of 3% peroxide. This was washed twice for 5 min in distilled water and dried. The slide was then covered with glycerol gelatin and a coverslip. The results were read visually through a microscope. The titer was expressed as a reciprocal of the highest dilution of the serum which demonstrated blue or blue black-colored rickettsial particles. We defined the serology diagnosis as positive when a ≥4-fold increase in the IP IgM/IgG titer against *R. japonica* or *O. tsutsugamushi* was observed in the paired samples or if the IP IgM titer was ≥320 in the acute phase sample. We excluded two cases, one who had a blood transfusion during treatment and another who was diagnosed with a concurrent infection of JSF and ST. We recorded the prefecture code of referred cases and defined the highest IgM titer of all serotypes as the responsible serotype in ST.

For statistical analysis, Spearman’s rank correlation coefficients were used between each *O. tsutsugamushi* serotype. The analysis was performed using STATA version 13.0 (Stata Corp, College Station, TX, USA). All patients’ data were anonymized for the analysis.

The current study was approved by the Institutional Review Board, Institute of Tropical Medicine, Nagasaki University.

## 3. Results

Of the 1406 cases examined during the study period, 621 cases had paired samples—154 of them were diagnosed as JSF and 138 were diagnosed as ST (Table 1): 148 JSF and 136 ST cases were diagnosed by a ≥4-fold increase of IgM/IgG titer in the paired samples, and 6 JSF and 2 ST cases were diagnosed by IgM ≥320 in the acute phase sample.

**Figure 1:**
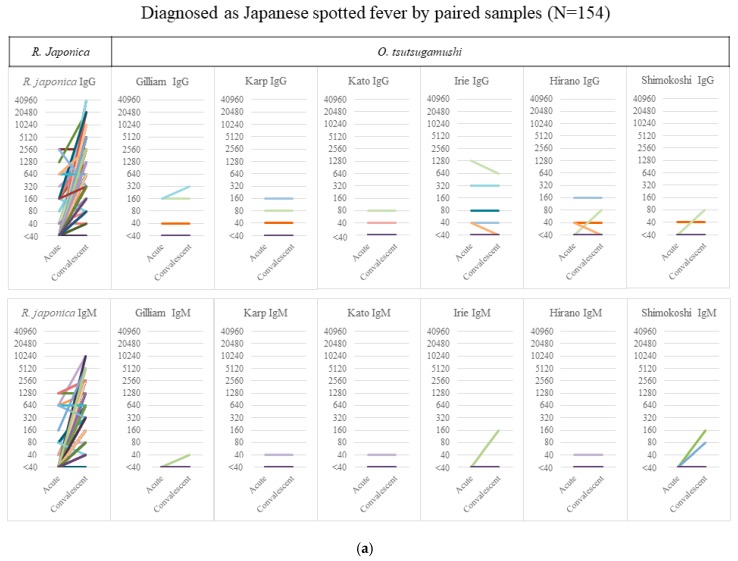

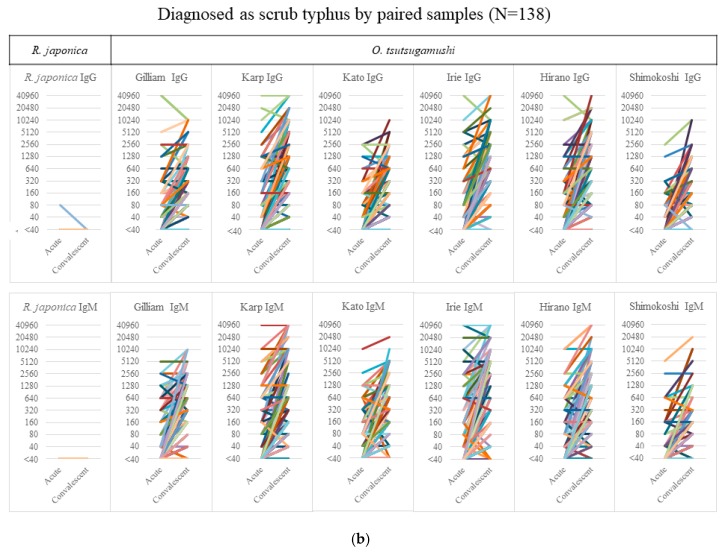
Serology results of cases of IgM/IgG against *R. japonica* and *O. tsutsugamushi.* The titer of IgM/IgG against *R. japonica* and the maximum titer of 6 serotypes against *O. tsutsugamushi* in Japanese spotted fever (**a**) and scrub typhus (**b**) are shown in acute and convalescent-phase samples. Abbreviations: Irie, Irie/Kawasaki; Hirano, Hirano/Kuroki.

From the acute to the convalescent phase, the median (interquartile range) duration of sample collection was 13 (8–17) days in JSF and 11 (7–15) days in ST. Figure 1a shows the serology results of 154 JSF cases—6 cases showed an IgG titer of ≥40 against *O. tsutsugamushi* without any significant elevation and 4 cases showed a nonsignificant IgM elevation of <320 in the paired samples, which had none/few cross-reactions with other *O. tsutsugamushi* serotypes. Figure 1b shows the serology results of 138 ST cases, none showing any rise in the IgM/IgG titer against *R. japonica* except for one with an IgG titer of 80 only in the acute phase sample. The figure, however, also shows a considerable degree of cross-reactivity between *O. tsutsugamushi* serotypes. It is noteworthy that the significantly high IgM titer of ≥320 against *R. japonica* was seen only in 9.7% (15/154) of JSF cases in the acute phase, whereas that against *O. tsutsugamushi* was seen in 73.2% (101/138) of ST cases.

Of the remaining 785 cases with only a single sample, 13 and 52 had significantly high IgM titers of ≥320 against *R. japonica* and *O. tsutsugamushi* in the acute phase sample, respectively (see Table 1), and none showed any cross-reactivity for IgM, although two ST cases had a low titer of IgG against *R. japonica* (data not shown).

**Figure 2:**
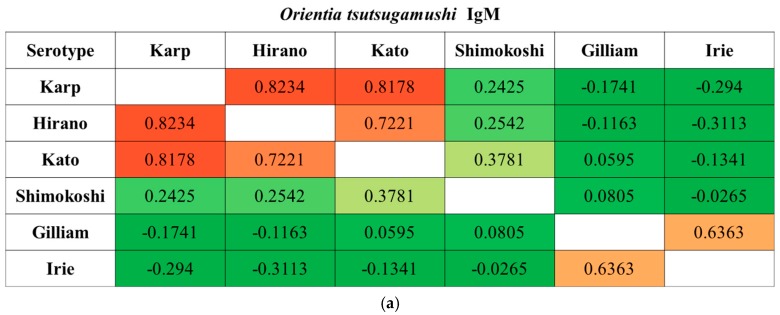

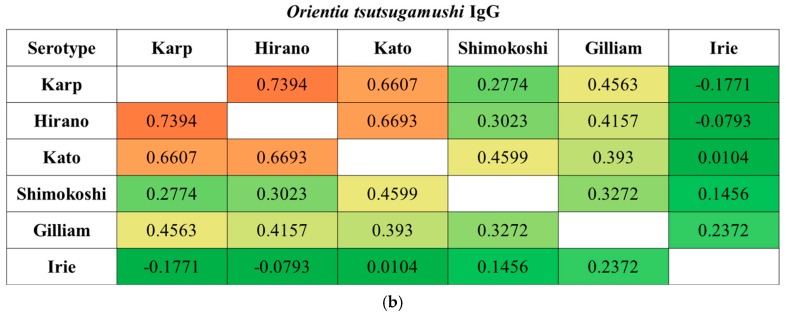
Spearman’s rank correlation coefficients between each *O. tsutsugamushi* serotype. The correlation coefficients in IgM (**a**) and IgG (**b**) samples were calculated using titers when a diagnosis of scrub typhus was made (*n* = 190): paired samples, *n* = 138; single sample, *n* = 52. Abbreviations: Irie, Irie/Kawasaki; Hirano, Hirano/Kuroki.

We observed a substantial degree of cross-reactivity between *O. tsutsugamushi* serotypes. When the correlation was further analyzed, cross-reactivity was significant among Karp, Hirano/Kuroki, and Kato types and between Gilliam and Irie/Kawasaki types in IgM when the diagnosis was made. In contrast, the Shimokoshi type was less cross-reactive than the others (Figure 2). This trend was also seen in IgG, except between Gilliam and Irie/Kawasaki types.

**Figure 3:**
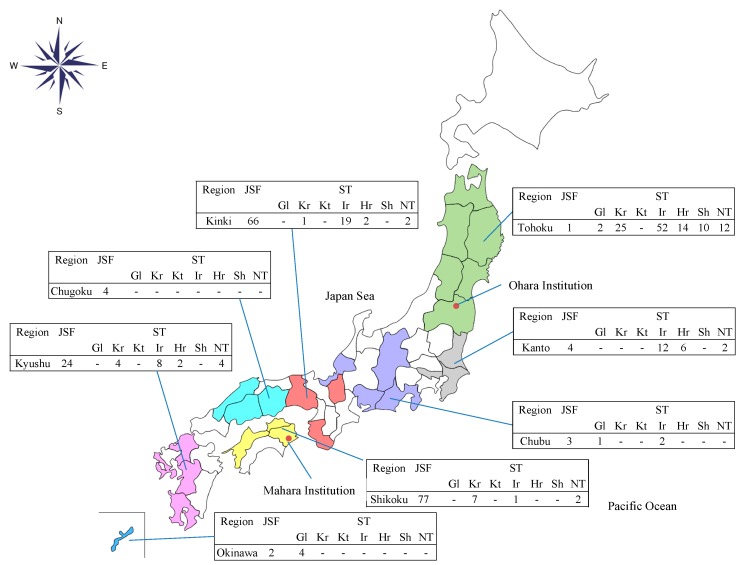
The number of cases serologically diagnosed as Japanese spotted fever or scrub typhus (serotypes) in each Japanese region at the reference centers. Abbreviation: JSF, Japanese spotted fever; ST, scrub typhus; Gl, Gilliam; Kr, Karp; Kt, Kato; Ir, Irie/Kawasaki; Hr, Hirano/Kuroki; Sh, Shimokoshi; NT, nontypeable; Ohara Institution, Ohara Research Laboratory, Ohara General Hospital; Mahara Institution, Mahara Institute of Medical Acarology.

The geographical distributions of the JSF and ST cases were different in that the JSF cases were diagnosed mainly among cases referred from western Japan, whereas the ST cases were diagnosed throughout the country, except for Hokkaido. Likewise, the pattern of *O. tsutsugamushi* serotypes showed a difference in geographical distributions (Figure 3). Gilliam and Karp types were dominant in Okinawa and Shikoku, respectively. Shimokoshi type was diagnosed only in Tohoku, and Irie/Kawasaki and Hirano/Kuroki were predominantly observed in Kanto, Kinki, and Kyushu.

## 4. Discussion

Our serology results demonstrate that the rickettsial diseases of *R. japonica* and *O. tsutsugamushi* do not serologically cross-react with each other, whereas a particular group of *O. tsutsugamushi* serotypes shows a considerable degree of cross-reactivity. *R. japonica* and *O. tsutsugamushi* have remarkable microbiological differences. Morphologically, the sizes of spotted fever and typhus group rickettsiae are smaller (in the range of 0.2–0.56 µm in width and 0.5–2.5 µm in length) than those found in *O. tsutsugamushi*, where the size of *O. tsutsugamushi* is in the range of 0.5–0.8 µm in width and 1.2–3.0 µm in length [4]. The genome size of *O. tsutsugamushi* is 2.0–2.6 megabase pairs, which is twice as large as that of other rickettsial species, including *R. japonica* (1.2–1.3 megabase pairs) [5,6]. The outer leaflet of the cell wall of spotted fever and typhus group rickettsiae is thinner than the inner leaflet, whereas the outer leaflet is thicker in *O. tsutsugamushi* [4]. *O. tsutsugamushi* lack peptidoglycan lipopolysaccharide and a capsule-like layer [7]. Further, all rickettsiae are susceptible to tetracyclines, chloramphenicol, and some other drugs. Although clinically, beta-lactams are inadvisable drugs for rickettsial diseases, in vitro studies have shown some effect for spotted fever and typhus group rickettsia but not for *O. tsutsugamushi* [4,8]. Its lack of lipopolysaccharide and peptidoglycan is compatible with the nonsusceptibility of *O. tsutsugamushi* to beta-lactams.

The sero-reactive antigens for spotted fever group rickettsiae and *Orientia* are different. The major serological antigens for spotted fever group rickettsiae are lipopolysaccharide and high-molecular-weight outer membrane proteins (rOmpA and rOmpB), whereas the most predominant antigen for *Orientia* is 56 kDa antigen [9,10]. Serological cross-reactions among rickettsial species, including *Rickettsia rickettsii*, *Rickettsia conorii*, and *Rickettsia typhi*, are known, although fewer cross-react with *R. typhi* [11]. Additionally, cross-reactions with TT-118 (=*Rickettsia honei*), *Rickettsia sibirica*, and *R. typhi* have been shown in JSF [2,12]. These cross-reactions appeared to be directed against lipopolysaccharide, suspected to contain similar epitopes (lacking in *O. tsutsugamushi*) in the IgM class [13]. Likewise, serological cross-reactions among different *O. tsutsugamushi* serotypes are also known [14], as shown in our results. However, it is noteworthy that some *O. tsutsugamushi* serotypes, such as Shimokoshi and Irie/Kawasaki types, have no cross-reaction/less cross-reaction with the standard serotypes [15,16].

Three JSF cases showed IgM titer ≥40 against *O. tsutsugamushi* in a nonsignificant low dilution. We think that this is a nonspecific reaction because of the absence or small amount of cross-reactivity with other *O. tsutsugamushi* serotypes in the convalescent phase sample. Additionally, the IgM against *O. tsutsugamushi* appeared only in low dilutions (≤160). The cutoff value for the diagnosis of ST may vary. In previous reports with a clear definition of the cutoff for ST diagnosis, one study with IP used ≥400 IgM and IgG [17], whereas another study with indirect immunofluorescence assays used a significant ≥4-fold rise to ≥200 in the convalescent phase sample or a single titer ≥400 [18]. Another study proposed a ≥3200 level of acute IgM to gain the optimal specificity [19]. In our study, since there has not been an established consensus of the cutoff, we presented all the results for which the titer was ≥40 to facilitate the discussion of cross-reaction. Furthermore, no IgM against *O. tsutsugamushi* was elevated in the acute phase sample, whereas the IgM was positive against *R. japonica*; we therefore concluded that there is no cross-reaction between *R. japonica* and *O. tsutsugamushi*.

The 6 JSF cases with high IgG titers against *O. tsutsugamushi* without elevation in the convalescent phase sample were suggestive of previous exposure to *O. tsutsugamushi*. Additionally, none of these 6 cases showed an IgM response, including nonspecific reactions. The JSF cases with previous exposure to *O. tsutsugamushi* or vice versa were rather rare, although both diseases are endemic in Japan. This may be because both diseases rarely had overlapping clusters at a regional level. Considering the variety of distributions and cross-reactivity among *O. tsutsugamushi* serotypes, we should carefully select a suitable combination of serotype antigens for each region, in addition to *R. japonica*.

Our geographical distribution data need to be interpreted with caution due to a selection bias. The reference system for rickettsial diseases has not been standardized in Japan, thus selection criteria to suspect rickettsial diseases may vary depending on the prefecture.

## 5. Conclusions

From the IP test, we concluded that no cross-reaction occurs between *R. japonica* and *O. tsutsugamushi*, whereas the cross-reactivity among *O. tsutsugamushi* occurs according to serotypes.

## Figures and Tables

**Table 1:** Summary of the tested cases for Japanese spotted fever and scrub typhus.

Total	Single-Sample Cases			Paired-Sample Cases				
	Tested	IgM ≥ 320 ^1^		Tested	≥4-Fold Increase ^2^		IgM ≥ 320 ^1^	
		JSF	ST		JSF	ST	JSF	ST
1406	785	13	52	621	148	136	6	2

^1^ The diagnoses were made by IgM ≥320 in the acute phase sample; ^2^ The diagnoses were made by a ≥4-fold increase of IgM/IgG titer in the paired samples. Abbreviations: JSF, Japanese spotted fever; ST, scrub typhus.
