# Correction: Sando E. et al. Serological Cross-Reactivity among *Orientia tsutsugamushi* Serotypes but Not with *Rickettsia japonica* in Japan. *Trop. Med. Infect. Dis.* 2018, 3, 74.

**DOI:** 10.3390/tropicalmed3040113

**Published:** 2018-10-25

**Authors:** Eiichiro Sando, Koya Ariyoshi, Hiromi Fujita

**Affiliations:** 1 Department of General Internal Medicine, Kameda Medical Center, Chiba 296-8602, Japan; 2 Department of Clinical Tropical Medicine, Nagasaki University Graduate School of Biomedical Sciences, Nagasaki 852-8523, Japan; kari@nagasaki-u.ac.jp; 3 Department of Clinical Medicine, Institute of Tropical Medicine (NEKKEN), Nagasaki University, Nagasaki 852-8521, Japan; 4 Mahara Institute of Medical Acarology, Tokushima 779-1510, Japan; fujitah7knu@y8.dion.ne.jp

The authors wish to make the following corrections to this paper [1]:

1. There are mistakes in this article about the percentage of positive IgM in JSF and ST because the number was based on the cut-off ≥40, not ≥320. On page 3, line 3–4, the sentence “high IgM titer of ≥320 against *R. japonica* was seen only in 9.7% (15/154) of JSF cases in the acute phase, whereas that against *O. tsutsugamushi* was seen in 73.2% (101/138) of ST cases” should be “high IgM titer of ≥320 against *R. japonica* was seen only in 5.2% (8/154) of JSF cases in the acute phase, whereas that against *O. tsutsugamushi* was seen in 47.1% (65/138) of ST cases”.
2. The authors of Reference [1] wish to replace Figure 3 with the following:

In this corrected figure, the number of each disease has changed in some areas because the number was based on the cut-off ≥40, not ≥320.

These changes have no material impact on the conclusions of our paper. The authors would like to apologize for any inconvenience caused to the readers by these changes.

## Figures and Tables

**Figure 3:**
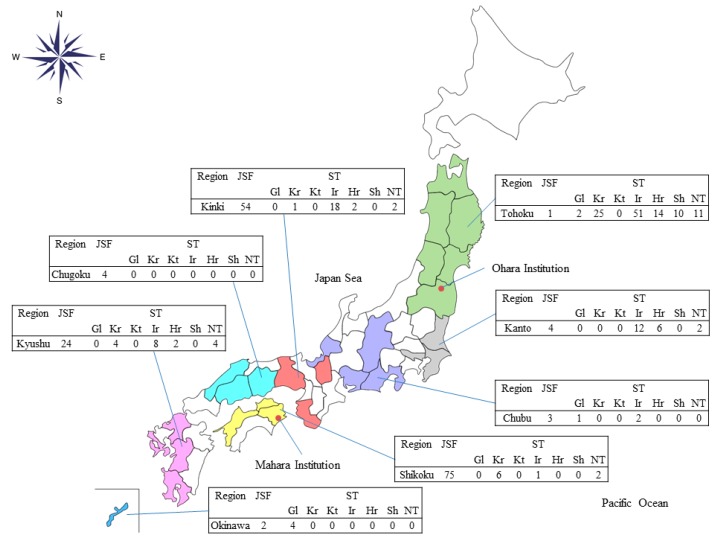
The number of cases serologically diagnosed as Japanese spotted fever or scrub typhus (serotypes) in each Japanese region at the reference centers. Abbreviation: JSF, Japanese spotted fever; ST, scrub typhus; Gl, Gilliam; Kr, Karp; Kt, Kato; Ir, Irie/Kawasaki; Hr, Hirano/Kuroki; Sh, Shimokoshi; NT, non-typeable; Ohara Institution, Ohara Research Laboratory, Ohara General Hospital; Mahara Institution, Mahara Institute of Medical Acarology.
